# Retrieving the Diurnal FPAR of a Maize Canopy from the Jointing Stage to the Tasseling Stage with Vegetation Indices under Different Water Stresses and Light Conditions

**DOI:** 10.3390/s18113965

**Published:** 2018-11-15

**Authors:** Liang Zhao, Zhigang Liu, Shan Xu, Xue He, Zhuoya Ni, Huarong Zhao, Sanxue Ren

**Affiliations:** 1 State Key Laboratory of Remote Sensing Science, Jointly Sponsored by Beijing Normal University and Institute of Remote Sensing and Digital Earth of Chinese Academy of Sciences, Beijing 100875, China; liangzhao@mail.bnu.edu.cn (L.Z.); bnuxushan@gmail.com (S.X.); xuehe@mail.bnu.edu.cn (X.H.); 2 Beijing Engineering Research Center for Global Land Remote Sensing Products, Institute of Remote Sensing Science and Engineering, Faculty of Geographical Science, Beijing Normal University, Beijing 100875, China; 3 Jiangxi Provincial Key Laboratory of Soil Erosion and Prevention, Jiangxi Institute of Soil and Water Conservation, Nanchang 330029, China; 4 Key Laboratory of Radiometric Calibration and Validation for Environment Satellites, National Satellite Meteorological Center, China Meteorological Administration, Beijing 100875, China; nizy@cma.gov.cn; 5 Chinese Academy of Meteorological Sciences, Beijing 100081, China; 656892rzr@163.com (H.Z.); Zhr680317@163.com (S.R.)

**Keywords:** FPAR, vegetation index, diurnal dynamics, drought, maize

## Abstract

The fraction of absorbed photosynthetically active radiation (FPAR) is a key variable in the model of vegetation productivity. Vegetation indices (VIs) that were derived from instantaneous remote-sensing data have been successfully used to estimate the FPAR of a day or a longer period. However, it has not yet been verified whether continuous VIs can be used to accurately estimate the diurnal dynamics of a vegetation canopy FPAR, which may fluctuate dramatically within a day. In this study, we measured the high temporal resolution spectral data (480 to 850 nm) and FPAR data of a maize canopy from the jointing stage to the tasseling stage under different irrigation and illumination conditions using two automatic observation systems. To estimate the FPAR, we developed regression models based on a quadratic function using 13 kinds of VIs. The results show the following: (1) Under nondrought conditions, although the illumination condition (sunny or cloudy) influenced the trend of the canopy diurnal FPAR, it had only a slight effect on the model accuracies of the FPAR-VIs. The maximum coefficients of determination (R^2^) of the FPAR-VIs models generated for the sunny nondrought data, the cloudy nondrought data, and all of the nondrought data were 0.895, 0.88, and 0.828, respectively. The VIs—including normalized difference vegetation index (NDVI), green NDVI (GNDVI), red-edge simple ratio (SR_705_), modified simple ratio 2 (mSR2), red-edge normalized difference vegetation index (NDVI_705_), and enhanced vegetation index (EVI)—that were related to the canopy structure had higher estimation accuracies (R^2^ > 0.8) than the other VIs that were related to the soil adjustment, chlorophyll, and physiology. The estimation accuracies of the GNDVI and some red-edge VIs (including NDVI_705_, SR_705_, and mSR2) were higher than the estimation accuracy of the NDVI. (2) Under drought stress, the FPAR decreased significantly because of leaf wilting and the effective leaf area index decrease around noon. When we included drought data in the model, accuracies were reduced dramatically and the R^2^ value of the best model was only 0.59. When we built the regression models based only on drought data, the EVI, which can weaken the influence of soil, had the best estimate accuracy (R^2^ = 0.68).

## 1. Introduction

The fraction of absorbed photosynthetically active radiation (FPAR) not only is an indicator that reflects the state of vegetation but also is an important input parameter of the vegetation productivity model [1]. The FPAR can be directly measured in the field by quantum sensors to gain the four independent photosynthetically active radiation (PAR) flux density components that account for the PAR entering and escaping from the plant canopy [2,3,4]. This method, however, is impractical to use for determining the FPAR over large areas because a large amount of quantum sensors would be required to account for the heterogeneity of the landscape [5]. Because remote sensing from orbiting platforms can repeatedly acquire consistent observations over large areas, satellite remote-sensing data have been widely used for FPAR estimation [6,7,8]. In general, methods of FPAR estimation from remote-sensing data are based on statistical or physical models [9]. The statistical models are mostly based on regression analysis between the ground-measured FPAR and vegetation indices (VIs) [9,10,11], whereas the physical models are based on canopy reflectance model inversion [6,7].

Substantial empirical evidence and radiative transfer model simulations show that the FPAR has a close relationship to the normalized difference vegetation index (NDVI) [10,12,13]. For example, Goward and Huemmrich (1992) found a nearly linear relationship between the NDVI and the daily FPAR [14]. Ridao, Conde, and Mínguez (1998) found that the VIs have a power function or exponential function relationship with the FPAR when the Leaf Area Index (LAI) is small and a linear relationship when the LAI increases [15]. The relationships between the NDVI and the FPAR, however, are not stable because of the impacts of sun/sensor geometry [16,17], substrate reflectance [11], and canopy architecture [18,19]. To overcome these impacts, many modified VIs or new VIs have been proposed to improve FPAR estimation. For example, the soil-adjusted vegetation index (SAVI), which incorporates an adjustment factor into the NDVI to account for the change in substrate reflectance, can obtain a stronger linear correlation with the FPAR than the NDVI can [16]. The green NDVI (GNDVI) and red-edge NDVI [20], which replace the red reflectance in the NDVI with the green reflectance and the red-edge reflectance, respectively, exhibit more sensitivity to the moderate-to-high green FPAR than does the NDVI [19,21]. The SAVI, which minimizes soil influences, exhibits a more linear relationship than does the NDVI [16]. A modification of the Chlorophyll Absorption in Reflectance Index (MCARI) was proposed [21] to minimize the effects of nonphotosynthetic materials on the spectral estimates of absorbed photosynthetically active radiation.

Various VIs derived from different satellite remote-sensing data have been used to estimate the FPAR successfully [10,22]. Because of the temporal resolution limitations of satellite remote-sensing data, most exiting studies have needed to use VIs from specific moments to estimate the FPAR of a longer period (e.g., several days or weeks) [10]. This method has been verified by the simulation results of the Scattering by Arbitrarily Inclined Leaves (SAIL) radiative transfer model. These results show that instantaneous NDVI measurements provide a stable and near-linear estimate of the diurnally integrated FPAR in an herbaceous vegetation canopy as long as the remotely sensed observations are taken when the solar zenith angle is above 60° and the sensor views are within 40° of the nadir [14]. Recent in situ measurements, however, show that the FPAR may fluctuate dramatically within a day because of changes in the light conditions [23] and stress states [24]. The FPAR diurnal course of an alpine wetland showed a “bowl-shaped” pattern, which was small around noon but larger in the early morning (before 10:00) and mid-afternoon (after 14:00) [23]. Under a water deficit, the decline of the FPAR around noon will become much more significant [24].

To estimate the FPAR more accurately, it is necessary to calculate the diurnal dynamics of the FPAR [23]. The improvement in the temporal resolution of remote-sensing data makes it possible to retrieve several VIs within a day. It remains to be studied, however, whether the VIs could be used to accurately estimate the diurnal variation of the FPAR using high temporal resolution.

To study this problem, we used an FPAR automatic observation system and a canopy spectral automatic observation system to obtain the long-term diurnal FPAR and the spectral data of a maize canopy. We used the spectral data to retrieve the VIs. We collected data under different light conditions and water stresses to study their influences on the relationship between the FPAR and the VIs.

## 2. Materials and Methods

### 2.1. Experimental Scheme

The study site (115°44′00″ E, 39°8′51″ N) was located at Gucheng, Baoding City, China. The experimental plot was a rectangular field of 2 × 4 m. To avoid the exchange of soil moisture, the plot was separated from the surrounding soil by concrete walls. A movable rain shelter was placed over this plot and the shelter was closed during rainfall to protect the maize. The soil moisture of the plot was controlled using artificial irrigation during the growth of the maize.

In this study, we continuously measured the maize canopy spectra and the photosynthetically active radiation (PAR) from 18 July to 3 August, 2017 (from the jointing stage to the tasseling stage), and removed rainy-day data (21–26 July). We collected data for 10 days (including sunny and cloudy days). The automatic observation time was from 7:00 to 18:00 every day. The detailed measurement methods and statistical methods are discussed in the following sections.

### 2.2. Canopy Hyperspectral Reflectance Data

We used an automated canopy spectral observation system that was improved based on a previous version [25] to collect the canopy spectral data. We used an uplooking CC-3 cosine-corrected irradiance probe (Ocean Optics Inc., Dunedin, FL, USA) to collect the downwelling irradiance. We used downlooking bare optical fibers with a field of view (FOV) of 25° to measure the upwelling radiance from the canopy at the nadir, approximately 2 m above the canopy. To collect the spectroscopy data, a spectrally and radiometrically calibrated spectrometer, QE65Pro (Ocean Optics Inc., Dunedin, FL, USA), was embedded in this system. The spectrum ranged from 480 to 850 nm and the spectral sampling interval was 0.4 nm.

### 2.3. Canopy FPAR

The canopy FPAR was collected automatically by a system named FPARNet (StarViewer, Beijing, China), which was revised from a previous version [26]. The system measured the total PAR reaching the top of the canopy (PARci), the PAR that was reflected back to the atmosphere (PARcr), the PAR that reached the soil through the canopy (PARgi), and the PAR that was reflected by the soil (PARgr). If the net PAR entering and exiting the canopy laterally was ignored, the FPAR was calculated with these components using the following formula [4]:

(1) $$\mathrm{FPAR}=\frac{{\mathrm{PAR}}_{\mathrm{ci}}-{\mathrm{PAR}}_{\mathrm{cr}}-\left({\mathrm{PAR}}_{\mathrm{gi}}-{\mathrm{PAR}}_{\mathrm{gr}}\right)}{{\mathrm{PAR}}_{\mathrm{ci}}}.$$

**Figure 1:**
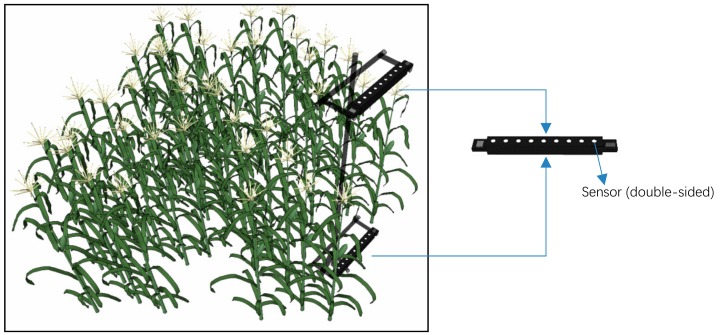
Automatic observation diagram of the FPAR.

Each component used average values calculated by nine independent sensors, which were evenly embedded into one side of a metal rod at intervals of 5 cm. Each variable in Equation (1) was measured by the two double-sided measuring rods that were placed horizontally at the top of the canopy and at the bottom of the canopy (approximately 15 cm from the ground) (Figure 1).

### 2.4. Soil Moisture

The relative moisture content (RMC), which is the percentage of soil moisture contained in the field water-holding capacities, can be calculated using the ratio of the weight moisture capacity (WMC) and the field water-holding capacity (WHC) (RMC = WMC/WHC). This value was measured before and after each irrigation. Soil samples (the sampling interval was 10 cm and the surface sampling depth as 5 cm) from different depths (the maximum depth was 1 m) were collected and weighed, and then the soil samples were placed in the oven at 105°C and dried for 24 h. The weight moisture capacity was calculated using the following formula [27]:

(2) $$\mathrm{WMC}=\frac{\mathrm{WS}-\mathrm{WD}}{\mathrm{WD}}$$

where WS is the weight of the wet soil and WD is the weight of the dry soil. The WMC that was calculated when the soil water content was saturated is represented by the WHC term. In this study, the WHC was 22.7.

### 2.5. Effective Leaf Area Index

When the soil moisture content is low and the light is strong, the leaves of maize will curl and the effective LAI will decrease [28]. Therefore, the effective LAI was measured to indicate the degree of drought stress. We measured LAI using an AccuPAR LP-80 (Decagon Devices, Inc., Pullman, WA, USA) from 10:00 to 16:00 at measurement intervals of 1.5 h [29]. The effective LAI was calculated using the following formula [30]:

(3) $${\mathrm{LAI}}_{\mathrm{eff}}=\frac{\left[\left(1-1/2K\right)\cdot f_{b}-1\right]\ln\tau }{A\cdot \left(1-0.47\cdot f_{b}\right)}$$

where τ is the hemispherically integrated transmittance, computed as the ratio of the below-canopy PAR and the incident PAR measured by the LP-80. f_b_ is the beam fraction (i.e., the ratio of the direct sunlight radiation to the total incoming radiation from all ambient sources). The beam fraction was automatically calculated with the instrument based on the latitude and the local time. K is the canopy extinction coefficient which was calculated with an assumption of a spherical leaf angle distribution and χ (leaf angle distribution parameter) was set to the default value of 1. A is a coefficient describing the canopy absorptivity and it is empirically related to the leaf’s absorptivity parameter. In this study, we adopted the default value (0.9) for the leaf absorptivity parameter.

### 2.6. Hyperspectral VIs

In reference to previous research [13,24,31,32,33,34], based on the characteristics of this study and combined with the physical meaning of the VI, we evaluated 13 VIs (Table 1) in relation to the canopy structure, chlorophyll, soil adjustment, and physiological response to stress. We also selected some red-edge VIs that had a good estimate effect for the FPAR, according to Dong et al. (2015) [31].

### 2.7. Data Fitting

Usually, the models of the FPAR-VIs are the linear and exponential types of models. The subject of study in this paper, however, is the diurnal FPAR. According to the curve characteristic of the diurnal FPAR, we adapted the quadratic equation to the FPAR as a function of the VIs in this study.

We evaluated the performance of the model through the coefficient of determination (R^2^) and the root mean squared error (RMSE) for the estimation of in situ–measured FPAR. The RMSE was calculated using Equation (4):

(4) $$\mathrm{RMSE}=\sqrt{\sum_{i=1}^{n}{\left(y_{m}-y_{p}\right)}^{2}/N},$$

where y_m_ and y_p_ represent the measured values and the predicted values, respectively; and the N term represents the number of samples.

## 3. Results

### 3.1. Soil Moisture and Effective LAI during Canopy Development

The relative moisture content in the shallow soil was 58.23% on 16 July (with irrigation on the evening of 14 July) and decreased to 36.92% on 30 July. After irrigating on the evening of 30 July, the relative moisture content of the shallow soil quickly returned to 56.83%. During this period, there was no significant change in the deep soil moisture. Irrigation had little effect on the deep soil moisture (50~100 cm), but it had an obvious effect on the shallow soil moisture (0–50 cm) (Table 2).

**Figure 2:**
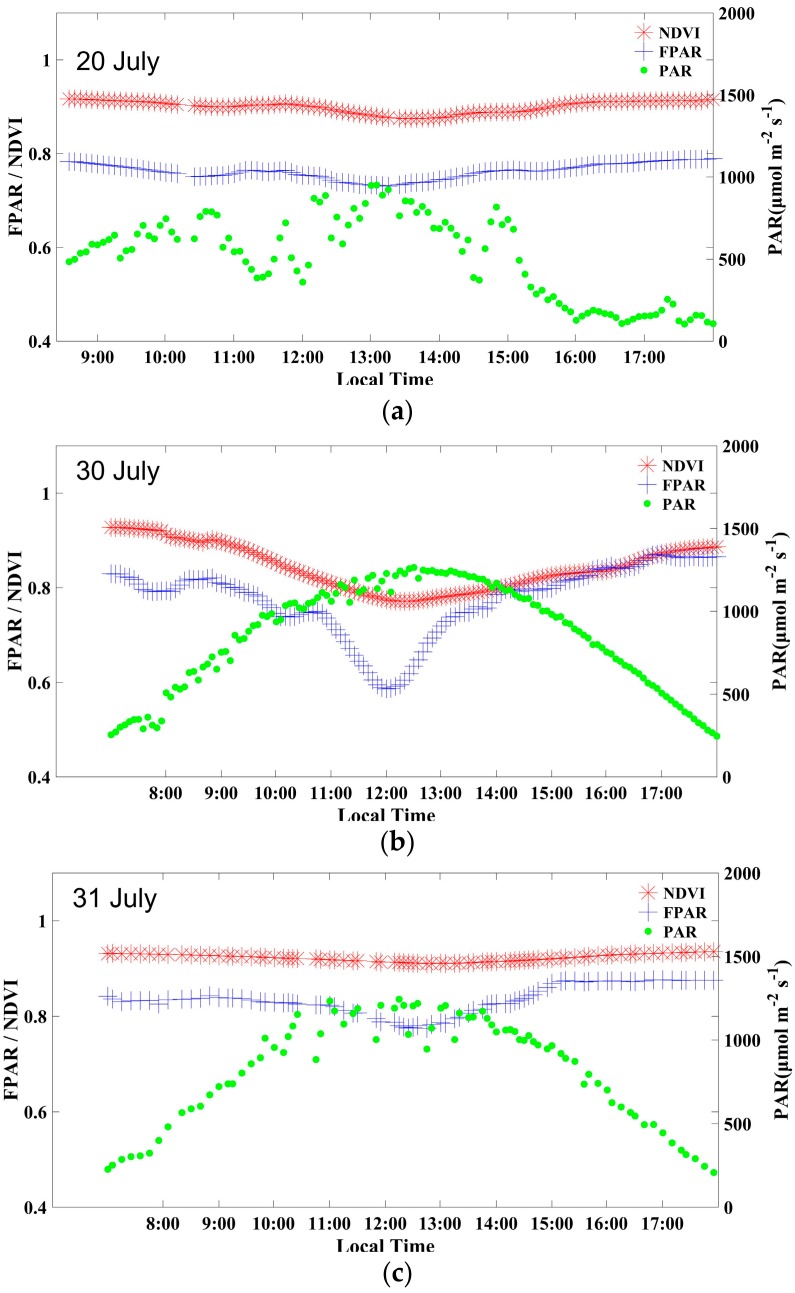
Diurnal FPAR, NDVI, and PAR during (**a**) a cloudy day without drought stress, (**b**) a sunny day with drought stress, and (**c**) a sunny day without drought stress.

The average effective LAI values on different days are shown in Table 3. After irrigation on 14 July, the average LAI showed a downward trend from 18 July to 31 July with the decrease in soil moisture. However, the average effective LAI on 27 July was an exception, as a result of leaf growth and stretching during the cloudy day. On 29 July and 30 July, the average effective LAI values were reduced significantly because the weather was sunny and the soil moisture was minimal, which led to severe curling of the leaves at noon (Figure 2b).

### 3.2. FPAR and NDVI under Different Conditions

The typical diurnal FPAR and PAR values are shown in Figure 2 under different conditions. As the most representative VI, the NDVI calculated from the spectral data is also shown. As the maize was under drought stress on 30 July, the curve of the diurnal FPAR had an obvious valley at approximately 12:00 (Figure 2b). This valley was due to the severe curl of the leaves at noon and the increase of light passing through the canopy to the ground. The maximum FPAR was found during the morning and afternoon, when the light intensity was weak and the leaves were not curled, although the soil moisture was low. The curve of the diurnal NDVI also had a valley at approximately 12:00, but the decrease was significantly smaller than that of the FPAR.

After the irrigation on the evening of 30 July, the average effective LAI increased from 2.12 on 30 July to 2.56 on 31 July. This change indicated that the wilting at noon disappeared with the increase in soil moisture. In this condition (sunny day), the diurnal canopy FPAR was mainly determined by changes in the solar zenith angle [35]. With the reduction of the solar zenith angle, the light saturation caused a decrease in the efficiency of the light used [36]. Therefore, at noon, the FPAR was lower than it was in the morning and afternoon. The decrease of the FPAR shown in Figure 2c was smaller than it was for the condition when the leaves were wilting shown in Figure 2b. In Figure 2c, however, the NDVI was almost constant.

On cloudy days, the incident light is mainly scattered light. The problem of the light saturation of vegetation does not occur at noon [37]. In this study, when the light intensity was low, the maize leaves did not curl at noon, even when the soil moisture was low. Therefore, on cloudy days, the diurnal dynamics of the FPAR and NDVI did not fluctuate significantly throughout the day (Figure 2a).

### 3.3. Retrieving the FPAR with VIs under Different Conditions

#### 3.3.1. Effect of Light Conditions on the Model Determination

To analyze the effect of light conditions on the model determination, we studied models built using the data from cloudy nondrought days and sunny nondrought days (Table 4). The results show that six kinds of VIs, including the NDVI, GNDVI, SR705, mSR2, NDVI_705_, and EVI, were optimal indices for estimating the diurnal FPAR under nondrought conditions. The R^2^ of the models built by these six VIs was greater than 0.8, whether using sunny- or cloudy-day data (Table 4). Changes in light conditions did not significantly influence the determination of these six FPAR-VI models under nondrought conditions.

Regardless of whether we used cloudy nondrought data or sunny nondrought data to construct the FPAR-VIs models, the fitting effect of the MCARI, which is related to chlorophyll (known as an important influence on the PAR absorbed by green vegetation), was always the worst. However, the fitting effect of the TCARI_705_, which is also related to chlorophyll, was better than that of the MCARI whether under sunny nondrought or cloudy nondrought conditions. The precision of the models built with the MSAVI, OSAVI, RDVI, RDVI_705_, and PRI was not ideal.

#### 3.3.2. Effect of a Drought on Model Accuracy

Using data from all days, drought days, and nondrought days, we built three kinds of statistical models between the FPAR and the 13 kinds of VIs (Table 5). When we used data from all days, the fitting effect of all the FPAR-VIs models was not ideal. It could be seen that fitting the FPAR with the GNDVI worked best, but the R^2^ value was only 0.590. The fitting effects of all FPAR-VIs models were significantly improved after removing the data from drought days. The R^2^ values of the six FPAR-VIs models, which were mentioned earlier, were all above 0.75. In addition, the models built by the other VIs had poor fitting effects.

When we used only the data from drought days, the fitting effect of all the FPAR-VIs models became worse than the fitting effect for the nondrought condition. Therefore, the introduction of the drought data led to a reduction in the model accuracy. This reduction was due to the FPAR being more sensitive to leaf wilting than the VIs at noon (Figure 2b). When the leaves wilted at noon, the effect of the soil on the VIs increased. Because the EVI can effectively eliminate the influence of the soil, the EVI had the highest correlation with the FPAR when using drought data to construct the model. The FPAR-EVI model that had the best model fit had an R^2^ value of only 0.685. The fitting effect of the PRI that had a poor performance under nondrought conditions significantly improved under drought conditions.

#### 3.3.3. Comparison of the Prediction Results of the Different Models

**Figure 3:**
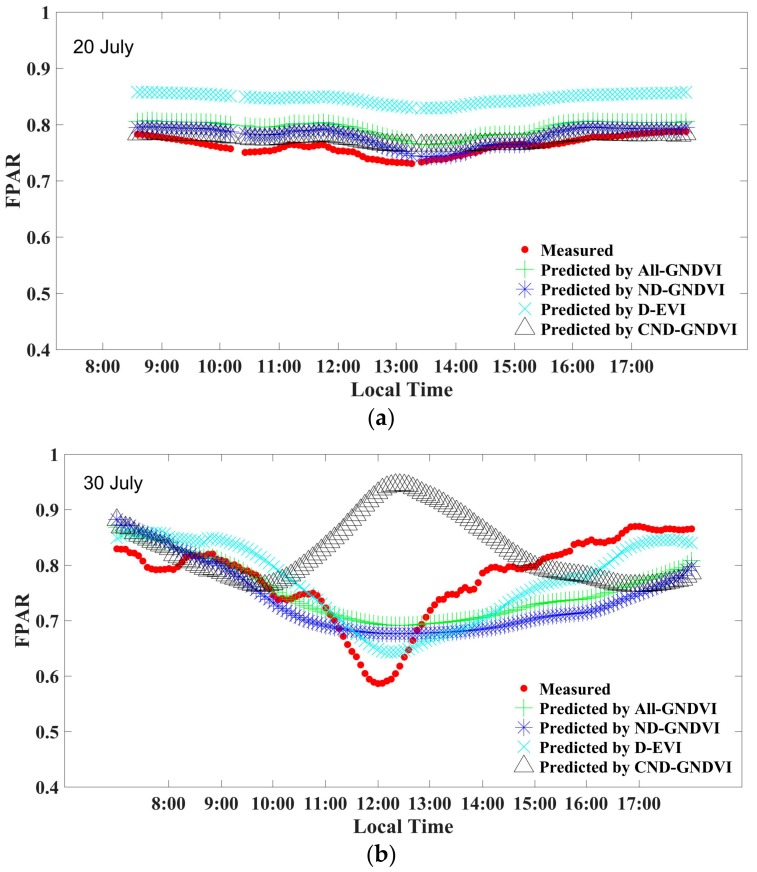

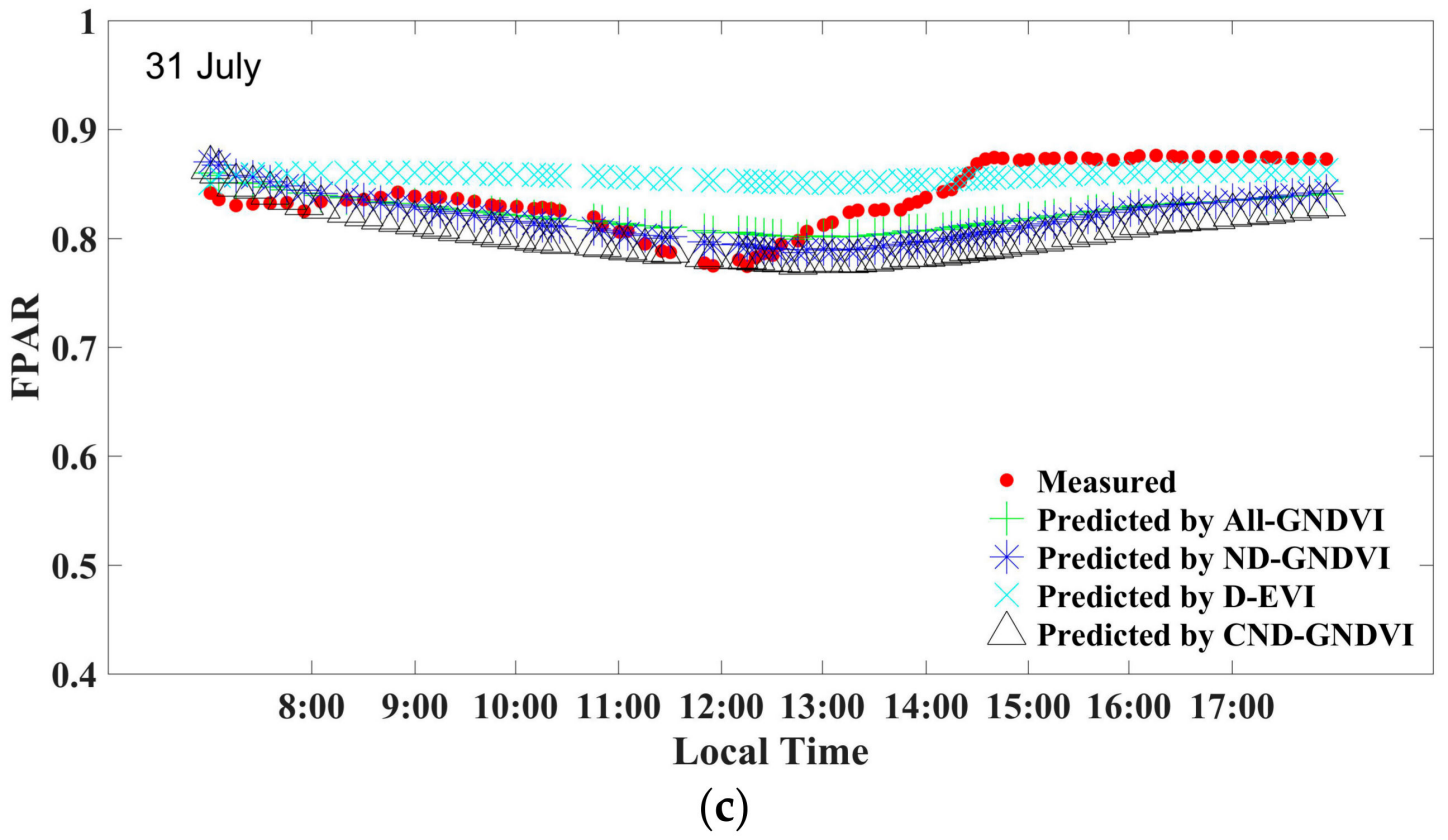
Measured FPAR and predicted FPAR of four models for (**a**) a cloudy nondrought day, (**b**) a sunny drought day, and (**c**) a sunny nondrought day.

To compare the prediction results of the models built using the different data, we selected the best example of each kind of model: (1) the model built using all of the data with the GNDVI (All-GNDVI), (2) the model built using all of the nondrought data with the GNDVI (ND-GNDVI), (3) the model built using the drought data with the EVI (D-EVI), and (4) the model built using the cloudy nondrought data with the GNDVI (CND-GNDVI). The prediction results for three typical days are shown in Figure 3 and given in Table 6.

The variation trends of the diurnal dynamics of the FPAR on a cloudy day (20 July) in the four models were all consistent with the measurement results. Except for the predicted high value of the D-EVI, the predicted value deviations of the other three models were very small (Table 6). When the drought caused the leaves to wither at noon (30 July), the predicted value of the CND-GNDVI had a significant error. The results of the other three models were similar, and the D-EVI had the highest accuracy compared with the other models. None of the other models, however, could predict the characteristics of a sudden sharp decrease in the FPAR at noon (Figure 3b). For the sunny day without drought stress data (31 July), the overall prediction accuracies of all four models were high (Table 6). The diurnal curve predicted by the D-EVI, however, did not reflect the slight decrease in the FPAR at noon. In general, the All-GNDVI had the highest accuracy and versatility for all three types of data.

## 4. Discussions

The overall trend of the diurnal FPAR in this study was consistent with that shown in a previously published article [23], but the trend of the diurnal FPAR was quite different under different environmental conditions. Most of the existing studies that estimate FPAR based on VIs have a low time resolution. This paper clearly showed the dynamic of diurnal FPAR under different environmental conditions.

Although VIs have been verified to be able to estimate daily or monthly FPAR, the change of environmental conditions can affect the physiological state of the vegetation, which leads to changes in the canopy FPAR during the day. Until now, no systematic and profound studies have evaluated the potential for VIs to estimate the dynamic of diurnal FPAR under different environmental conditions. Studying the diurnal canopy FPAR under different conditions may help verify and improve the accuracy of FPAR estimation using the data from a single satellite transit [23], and it can provide a way to better understand the change of the vegetation physiological state.

When we used data from the nondrought days, the estimation accuracy of the diurnal FPAR used the most VIs (except RDVI and RDVI_705_) that were related to the canopy structure, and this accuracy was higher than that of the other VIs that were related to soil adjustment, chlorophyll, and physiology (Table 4 and Table 5). Because the effective LAI was large in the study period, the soil background was not a main factor affecting the canopy diurnal FPAR. The models built using VIs (MSAVI, OSAVI) that were related to soil adjustment did not have a good fitting effect. In addition, the pigment content and physiological state of the vegetation did not changed much within one day in nondrought condition. Thus, the models built by VIs (i.e., TCARI_705_, MCARI, and PRI) that were related to chlorophyll and physiology also did not have a good fitting effect.

The estimation accuracies of the GNDVI (Table 5) and some red-edge VIs (including NDVI705, SR705, and mSR2) were slightly lower than those of the NDVI. This consequence may have been a result of the saturation effects on the NDVI and the fact that the green- and red-edge reflectances are more sensitive than the red reflectance to the FPAR variation of a canopy with a high biomass [5,20,38,39]. This was consistent with the research results of Dong et al. (2015) [31].

Under drought conditions, the model built using the EVI had the best fitting effect comparison with other VIs that were related to canopy structure because the effective LAI decreased (see Section 2.3 and Table 2) and the curled leaves led to an increase of the bare soil background. An EVI can weaken the influence of soil background [34]. Therefore, the estimation accuracy of the EVI in drought conditions was the highest. However, the models built using the other two VIs (OSAVI, MSAVI) that were related to soil adjustment did not have a good fitting effect.

Most VIs that are related to the FPAR are calculated using the bands from visible light to near infrared. In this study, the spectral range of the spectrometer was limited to 480~850 nm. We did not evaluate some of the VIs based on the shortwave blue band. It is necessary to select more vegetation indices to conduct a broader comparative study in the future. Additionally, the data in the study was collected during the period from the jointing stage to the tasseling stage. The dynamic range of the diurnal FPAR during a day was small and the FPAR was high, even in the case of drought (the minimum value of the FPAR was about 0.6). The period of the large dynamic range of the diurnal FPAR (prevegetation growth period) needs to be studied further. The vegetation type selected in this study was only the canopy of summer maize. Retrieving the diurnal FPAR of other canopies (such as wheat, soybean) is a subject for further exploration.

## 5. Conclusions

In this study, we automatically measured the canopy diurnal reflectance data and FPAR data for summer maize from the tassel stage to the flowering stage and from the jointing stage to the tasseling stage to study the potential of retrieving the diurnal dynamics of the FPAR with 13 kinds of VIs under different irrigation and illumination conditions. We adapted the quadratic function model to compare the fitting effect of the FPAR-VIs under different conditions. The following conclusions were reached:

1. The influence of the illumination change on the effect of the FPAR-VIs models was not significant. The maximum coefficients of determination (R^2^) of the FPAR-VIs models generated by the sunny nondrought data, the cloudy nondrought data, and all of the nondrought data were 0.895, 0.88, and 0.828, respectively. The VIs (including NDVI, GNDVI, SR_705_, mSR2, NDVI_705_, and EVI) that were related to the canopy structure had a higher estimation accuracy (R^2^ > 0.8) than the other VIs that were related to the soil adjustment, chlorophyll, and physiology. The estimation accuracies of the GNDVI and some red-edge VIs (including NDVI_705_, SR_705_, and mSR2) were higher than those of the NDVI.
2. Drought greatly reduced the accuracy of the FPAR-VI models. When we compared the quadratic VI-FPAR models under drought and normal conditions in the maize canopy, the maximum R^2^ value for the quadratic FPAR-VI models built using all of the data (including the drought data) was only 0.590. The maximum R^2^ value was 0.828 for the quadratic VI-FPAR models after eliminating the drought data. When we built the regression models based on only the drought data, the EVI had a better performance in estimating the diurnal canopy FPAR than the other VIs that were related to the canopy structure.
3. The quadratic models for the VIs were suitable for the prediction of the FPAR under nondrought conditions. No quadratic models of VIs could predict the characteristics of a sudden sharp decrease in the FPAR at noon under drought stress. Further research is required to develop a power model (e.g., a higher-order polynomial model) between the FPAR and the VIs to predict the diurnal dynamics of the FPAR under drought stress.

## Figures and Tables

**Table 1:** Definitions of the vegetation indices (VIs) evaluated in this study.

Name	Index	Formulation
Re-normalized difference vegetation index	RDVI	(R_800_ − R_670_)/($\sqrt{R_{800}+R_{670}}$)
Enhanced vegetation index	EVI	2.5 × (R_800_ − R_690_)/(R_800_ + 6.0 × R_690_ − 7.5 × R_490_)
Green normalized difference vegetation index	GNDVI	(R_800_ − R_550_)/(R_800_ + R_550_)
Modified soil-adjusted vegetation index	MSAVI	(2 × R_800_ + 1 − $\sqrt{{\left(2\times R_{800}+1\right)}^{2}-8\times \left(R_{800}-R_{670}\right)}$)/2
Normalized difference vegetation index	NDVI	(R_800_ − R_670_)/(R_800_ + R_670_)
Red-edge simple ratio	SR_705_	R_750_/R_705_
Modified simple ratio 2	mSR2	(R_750_/R_705_ − 1)/($\sqrt{R_{750}/R_{705}+1}$)
Red-edge normalized difference vegetation index	NDVI_705_	(R_750_ − R_705_)/(R_750_ + R_705_)
Optimal soil-adjusted vegetation index	OSAVI	(1 + 0.16) × (R_800_ − R_670_)/(R_800_ + R_670_ + 0.16)
Red-edge re-normalized difference vegetation index	RDVI_705_	(R_800_ − R_705_)/($\sqrt{R_{800}+R_{705}}$)
Red-edge transformed chlorophyll absorption in reflectance index	TCARI_705_	3 × [(R_750_ − R_705_) − 0.2 × (R_750_ − R_550_) × (R_750_/R_705_)]
Modified chlorophyll absorption in reflectance index	MCARI	((R_700_ − R_670_) − 0.2 × (R_700_ − R_550_)) × (R_700_/R_670_)
Photochemical reflectance index	PRI	(R_531_ − R_570_)/(R_531_ + R_570_)

**Table 2:** Weight moisture capacity (WMC) and relative moisture content (RMC) at different depths (Irrigation was carried out on the nights of 14 July and 30 July).

Depth (cm)	16 July		30 July		1 August	
	WMC	RMC	WMC	RMC	WMC	RMC
0~5	14.3	58.23%	3.0	36.92%	14.4	56.83%
5~10	13.7		6.2		14.1	
10~20	12.7		8.9		14.6	
20~30	10.2		9.1		8.3	
30~40	15.3		14.7		13.1	
40~50	16.7		16.5		15.4	
50~60	17.3	69.34%	17.0	69.02%	14.4	62.63%
60~70	15.9		16.1		13.8	
70~80	15.1		15.0		13.5	
80~90	14.2		13.8		13.9	
90~100	15.2		15.6		14.3	

**Table 3:** The average effective LAI values on different days.

Date	Effective LAI	Weather	Date	Effective LAI	Weather
18 July 2017	2.65	Sunny	29 July 2017	2.08	Sunny
19 July 2017	2.4	Sunny	30 July 2017	2.12	Sunny
20 July 2017	2.21	Cloudy	31 July 2017	2.56	Sunny
27 July 2017	2.46	Cloudy	1 August 2017	3.31	Sunny
28 July 2017	2.28	Cloudy	3 August, 2017	2.99	Sunny

**Table 4:** Regression equations between the FPAR (y) and VIs (x) on sunny days and cloudy days without drought stress.

Cloudy Nondrought Days				Sunny Nondrought Days			
VIs	Formula	R^2^	RMSE	VIs	Formula	R^2^	RMSE
GNDVI	y = 35.3025x^2^ − 54.0546x + 21.4547	0.880	0.014	mSR2	y = −0.0506x^2^ + 0.5376x − 0.0347	0.895	0.015
SR_705_	y = 0.0227x^2^ − 0.2325x + 1.3599	0.873	0.014	SR_705_	y = −0.0073x^2^ + 0.1723x + 0.0288	0.895	0.014
mSR2	y = 0.4326x^2^ − 1.4608x + 1.9994	0.872	0.014	GNDVI	y = −6.435x^2^ + 12.8143x − 5.301	0.889	0.015
NDVI_705_	y = 16.9178x^2^ − 22.9836x + 8.5701	0.867	0.014	NDVI	y = 23.2133X^2^ − 39.6909x + 17.6786	0.889	0.015
NDVI	y = 24.69x^2^ + −43.4435x + 19.8771	0.844	0.016	NDVI_705_	y = 6.0087x^2^ − 6.5581x + 2.4183	0.888	0.015
EVI	y = 2.4251x^2^ − 9.9697x + 11.0135	0.833	0.016	EVI	y = 2.2449x^2^ − 8.8946x + 9.5286	0.857	0.017
TCARI_705_	y = 0.0033x^2^ − 0.0244x + 0.8056	0.799	0.018	TCARI_705_	y = −0.0022x^2^ + 0.0618x + 0.4854	0.626	0.028
MSAVI	y = 6.5224x^2^ − 10.4731x + 4.9721	0.789	0.018	MSAVI	y = 2.0351x^2^ − 2.4975x + 1.4337	0.611	0.028
OSAVI	y = 9.2741x^2^ − 14.9208x + 6.7686	0.771	0.019	OSAVI	y = −1.8846x^2^ + 4.5102x − 1.6842	0.568	0.030
RDVI_705_	y = 3.7319x^2^ − 4.1813x + 1.9315	0.765	0.019	RDVI_705_	y = −2.6621x^2^ + 4.2621x − 0.8434	0.473	0.033
PRI	y = 51.7959x^2^ + 1.2097x + 0.775	0.756	0.020	RDVI	y = −3.3692x^2^ + 5.7419x − 1.6118	0.384	0.036
RDVI	y = 3.0198x^2^ − 3.9242x + 2.0366	0.713	0.021	PRI	y = −40.9556x^2^ + 3.5204x + 0.7674	0.363	0.036
MCARI	y = 1.11x^2^ + 1.7204x + 0.6741	0.595	0.025	MCARI	y = −73.7611x^2^ + 13.8129x + 0.1759	0.360	0.036

**Table 5:** Regression equations between the FPAR (y) and VIs (x) for three different kinds of conditions.

18 July to 3 August (All Days)				18 July to 3 August (Nondrought Days)				18 July to 3 August (Drought Days)			
VIs	Formula	R^2^	RMSE	VIs	Formula	R^2^	RMSE	VIs	Formula	R^2^	RMSE
GNDVI	y = 4.9269x^2^ − 6.1141x + 2.5617	0.590	0.036	GNDVI	y = 10.201x^2^ − 13.8935x + 5.4054	0.828	0.018	EVI	y = −0.3955x^2^ + 1.833x − 1.2591	0.685	0.044
SR_705_	y = 0.0034x^2^ + 0.0038x + 0.658	0.549	0.038	NDVI_705_	y = 7.51x^2^ − 9.189x + 3.5267	0.813	0.018	NDVI	y = −7.5738x^2^ + 14.2627x − 5.8525	0.654	0.046
mSR2	y = 0.073x^2^ − 0.0915x + 0.7175	0.547	0.038	NDVI	y = 24.4468x^2^ − 42.6451x + 19.3503	0.811	0.019	GNDVI	y = −12.0055x^2^ + 19.859x − 7.3489	0.653	0.046
NDVI_705_	y = 2.7775x^2^ − 2.8914x + 1.4561	0.536	0.039	mSR2	y = 0.0852x^2^ − 0.0673x + 0.6179	0.807	0.019	NDVI_705_	y = −4.2147x^2^ + 6.4723x − 1.6216	0.645	0.046
EVI	y = 0.281x^2^ − 0.8641x + 1.3689	0.500	0.040	SR_705_	y = 0.0011x^2^ + 0.0483x + 0.4642	0.805	0.019	mSR2	y = −0.1857x^2^ + 0.8028x − 0.0022	0.621	0.048
NDVI	y = 3.8763x^2^ − 5.7719x + 2.8513	0.495	0.040	EVI	y = 2.6888x^2^ − 11.011x + 12.0306	0.790	0.019	SR_705_	y = −0.0129x^2^ + 0.1845x + 0.2076	0.602	0.049
TCARI_705_	y = 0.0006x^2^ + 0.0124x + 0.7002	0.427	0.043	TCARI_705_	y = 0.0007x^2^ + 0.0159x + 0.6604	0.662	0.025	PRI	y = −34.9698x^2^ + 0.8984x + 0.8607	0.580	0.050
MSAVI	y = 1.7611x^2^ − 2.3618x + 1.5346	0.398	0.044	MSAVI	y = 5.0591x^2^ − 7.8632x + 3.8112	0.658	0.025	OSAVI	y = −5.0779x^2^ + 8.9373x − 3.0722	0.522	0.054
OSAVI	y = 2.0062x^2^ − 2.6163x + 1.5778	0.395	0.044	OSAVI	y = 5.6676x^2^ − 8.6318x + 4.0299	0.624	0.026	TCARI_705_	y = −0.0037x^2^ + 0.0641x + 0.595	0.481	0.057
RDVI_705_	y = 0.8962x^2^ − 0.5841x + 0.8075	0.378	0.045	RDVI_705_	y = 0.6643x^2^ − 0.1069x + 0.586	0.581	0.028	MSAVI	y = −1.785x^2^ + 3.2967x − 0.663	0.461	0.057
RDVI	y = 0.8471x^2^ − 0.7532x + 0.8937	0.325	0.047	PRI	y = 42.5846x^2^ + 1.5045x + 0.7736	0.506	0.030	RDVI_705_	y = −2.8878x^2^ + 4.0519x − 0.5601	0.459	0.058
PRI	y = 6.8559x^2^ + 1.5202x + 0.7968	0.322	0.047	RDVI	y = 0.6001x^2^ − 0.2575x + 0.6511	0.500	0.030	RDVI	y = −2.5379x^2^ + 4.0916x − 0.7887	0.410	0.060
MCARI	y = 5.9394x^2^ + 0.6518x + 0.7253	0.216	0.050	MCARI	y = 4.0202x^2^ + 1.1067x + 0.693	0.375	0.034	MCARI	y = −26.3964x^2^ + 5.7026x + 0.5679	0.242	0.068

**Table 6:** The RMSE values for the predicted FPAR and the measured FPAR.

	All-GNDVI	ND-GNDVI	D-EVI	CND-GNDVI
**20 July**	0.033	0.020	0.085	0.017
**30 July**	0.063	0.075	0.049	0.134
**31 July**	0.031	0.034	0.034	0.044
